# MACHINE LEARNING APPROACHES FOR FALL PREDICTION IN KOREAN COMMUNITY-DWELLING OLDER ADULTS

**DOI:** 10.1093/geroni/igae098.3368

**Published:** 2024-12-31

**Authors:** Jungwon Cho, Minhee Yang, Eunhee Cho

**Affiliations:** Yonsei University, Seoul, Republic of Korea; Yonsei University, Seoul, Republic of Korea; Yonsei University, Seoul, Republic of Korea

## Abstract

Falls cause significant physical, psychological, and financial burdens for older adults. They represent the second leading cause of death worldwide. On average, approximately 27% of older adults fall annually. To prevent fall-related injuries, identifying older adults at risk for falls and engaging them in fall-prevention interventions are important measures. This study aimed to develop and evaluate the fall prediction model for older adults in the community with machine learning approach. A total of 9,884 participants were included in the analysis from the 2020 Korean National Survey of Older Adults. The dataset included 48 predictor variables, including demographics, health status, health behavior, and environmental factors. We employed several prediction models, including logistic regression, decision tree, random forest, and support vector machine. Synthetic minority oversampling technique (SMOTE) approaches were employed to address class imbalance. The area under the receiver operating characteristic curve (AUC) ranged from 0.84 to 0.98 across four algorithms. The random forest model had the highest AUC value of 0.98, followed by logistic regression (0.91), support vector machine (0.91), and decision tree (0.84). Marital status, the presence of visual impairment, economic activity status, sex, and diagnosis of hypertension were the five most important features for predicting falls. Our findings suggest that machine learning methods can accurately identify older adults at a high risk for falls, potentially enabling early interventions and targeted preventive measures. This approach could ultimately lead to reductions in fall-related injuries and healthcare costs and improvements in the overall quality of life of older adults.

